# Attitudes of older adults with serious competing health risks toward their implantable cardioverter-defibrillators: a pilot study

**DOI:** 10.1186/s12877-015-0173-2

**Published:** 2015-12-23

**Authors:** Ariel R. Green, Cynthia M. Boyd, John Rickard, Robert Gomon, Bruce Leff

**Affiliations:** Department of Medicine, Division of Geriatric Medicine and Gerontology, Johns Hopkins University School of Medicine, 5200 Eastern Avenue, 7th floor, Baltimore, MD 21224 USA; Department of Medicine, Division of Cardiology, Johns Hopkins University School of Medicine, 4940 Eastern Avenue, 301 building, Baltimore, MD 21224 USA; Department of Health Policy and Management, Johns Hopkins University Bloomberg School of Public Health, Baltimore, USA; Department of Community and Public Health, Johns Hopkins School of Nursing, Baltimore, USA

**Keywords:** Implantable cardioverter-defibrillator, Multimorbidity, Decision making

## Abstract

**Background:**

In elderly heart failure patients, the survival benefit of implantable cardioverter-defibrillators (ICDs) may be attenuated due to competing health risks, and the risk of adverse outcomes magnified. Our objective was to examine older adults’ attitudes towards ICD implantation in the context of competing health risks, exploring the determinants of ICD decision-making among a group of patients who had faced the decision in the past.

**Methods:**

Telephone survey with a qualitative component. Patients were age ≥70 with single- or dual-chamber ICDs from a single academic cardiac device clinic. Health status was assessed with the Vulnerable Elders Survey (VES-13). Responses to open-ended questions were transcribed verbatim; an “editing analysis” approach was used to extract themes.

**Results:**

Forty-four ICD recipients participated (mean age 77.5 years). Nineteen participants (43 %) had VES-13 scores ≥3, indicating a 50 % likelihood of death or functional decline within 2 years. Twenty-one participants (48 %) had received prior ICD shocks. Forty participants (91 %) said they would “definitely” choose to get an ICD again in their current health. By and large, patients revealed a strong desire to extend life, expressed complete confidence in the lifesaving capabilities of their ICDs, and did not describe consideration of competing health risks.

**Conclusions:**

In this pilot telephone survey with a qualitative component, nearly all older adults with ICDs would still choose to get an ICD despite high short-term risk of death or health deterioration. These findings suggest the need to partner more effectively with patients and families to decide how best to use medical technologies, particularly for older adults with competing risks.

## Background

Despite a lack of conclusive evidence of effectiveness for older adults, over 40 % of the 110,000 implantable cardioverter-defibrillators (ICDs) implanted in the U.S. each year are in patients over age 70, and 10 to 20 % are in patients over age 80 [1–3]. Primary prevention ICD clinical trials demonstrated no survival advantage for the first 9–18 months after implantation [4, 5]. The 1-year mortality rate for a 75-year-old patient hospitalized with heart failure and low ejection fraction is 30–50 %, and fewer than 10 % of deaths in this population are due to sudden cardiac death [6]. Thus, many of these deaths are due to competing health risks.

ICDs are a prophylactic therapy that carry risks: post-procedural complications, an increase in hospitalizations, inappropriate shocks, and futile end-of-life shocks among patients dying of non-arrhythmic causes [7–9]. Furthermore, ICDs (in the absence of cardiac resynchronization therapy, or CRT) do not affect heart failure symptoms, and their effect on quality of life is, at best, neutral. However, data suggest that patients may consent to implantation with the expectation that their physical health and functioning will improve [10]. This misconception is particularly important for older adults with multiple coexisting illnesses, in whom ICDs are unlikely to result in a survival benefit, and may make life and death more onerous [11–13].

We hypothesized that older adults considering ICD therapy do not weigh their competing risks for mortality, and that this unrealistic approach leads to ICD implantation in situations where there may be harms without benefit. Our objective was to examine older adults’ attitudes towards ICD implantation in the context of competing health risks, exploring the determinants of ICD decision-making among a group of patients who had faced the decision in the past.

## Methods

### Study design, setting and participants

This was a cross-sectional telephone survey and qualitative study of older adults (≥70 years) with ICDs placed for either primary or secondary preventive purposes, who were actively being followed in the cardiac device clinic of Johns Hopkins Bayview Medical Center, an academic medical center. Patients were identified from the clinic database.

**Fig. 1:**
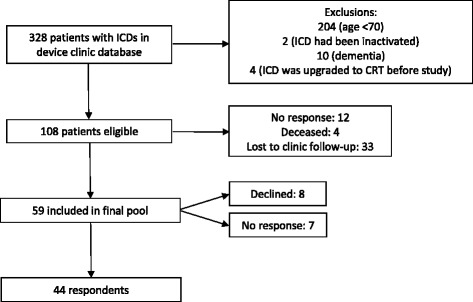
Patients were identified from the clinic database. Eligible participants were English-speaking and had a single or dual-lead ICD (not capable of CRT)

Eligible participants were English-speaking and had a single or dual-lead ICD (not capable of CRT). We excluded patients who had dementia or lived in a nursing home, based on a family member’s report (Fig. 1), because they may lack capacity to make medical decisions. We also excluded patients whose ICDs had been deactivated, because we wanted to probe determinants of ICD decision-making among patients who were likely to face the decision again in the future.

One investigator (RG) identified potential participants and sought their approval to be contacted about the study by telephone. Those who agreed were contacted by the principal investigator (AG), a geriatrician with experience conducting research interviews. She explained the purpose and procedures of the study in detail, obtained verbal informed consent, and administered a 15-min telephone survey with a qualitative component. After giving informed consent, participants completed the survey during the same phone call. Surveys were completed between April and November 2013. The study was approved by the Johns Hopkins University School of Medicine Institutional Review Board.

### Survey development and administration

The pilot study consisted of a 29-question survey on demographics, medical history, functional status, perceptions of ICD benefit and burdens, personal experiences with the ICD, and health literacy. The medical history questions included geriatric impairments such as urinary or fecal incontinence, difficulty walking, frequent falls, dizziness, depression or sadness, and problems with hearing or vision.

To identify participants at increased risk of death or functional decline within 2 years, we used the Vulnerable Elders Survey (VES-13) [14]. The VES-13 is a 13-question screen that uses self-reported health and functional status measures to predict health deterioration. The survey was developed among 6205 nationally representative, community-dwelling Medicare beneficiaries ≥65 years, and has been validated in other populations of older adults [15–18]. Fifty percent of older adults with a score of ≥3 experience functional decline (defined as change from no functional disability to any functional disability, an increase of two or more in the total disability count, or admission to a nursing home) or die in a 2-year period and have 4.2 times the risk of death or decline, compared with those with scores <3 [14].

Perceptions of ICD benefit and burdens were assessed by asking participants, “In your current health state, do you feel that the potential benefit of your ICD is worth the burdens?” and “In your current health state, if you had the decision to make over again, would you still choose to get an ICD?” The latter question was adapted from the Decision Regret Scale, a questionnaire for measuring regret after health care decisions [19]. Participants responded using a Likert scale ranging from 1 (“definitely no”) to 5 (“definitely yes”) and were then asked to provide an open-ended explanation. The survey also included two additional open-ended questions: “What do you feel are the potential benefits of your ICD?” and “What do you feel are the potential harms of your ICD?” [20].

Participants were asked if they had experienced any device activity or complications, and if they thought their ICD had saved their life (“yes,” “no,” or “not sure”). We also assessed their understanding of ICD survival benefit and function, including device deactivation, using multiple-choice questions adapted from prior studies [21]. Because limited health literacy may be associated with worse health outcomes, poorer knowledge about health conditions and poorer self-reported health, we assessed health literacy with a 3-item screening tool with established validity [22]. Using established methods, we assigned 0 (no problems with reading) to 4 points (highest problems with reading) to the responses for each question. Scores for the 3 questions were combined to obtain a 12-point scale, with 0 = no problems with health literacy and 12 = highest problems with health literacy [22].

### Outcome

The main outcome was the proportion of participants who would still get an ICD in their current health state, if they had the choice to make again.

### Analysis

We used descriptive statistics to explore baseline characteristics of the population. The VES-13 was scored using a range of 1–10 with higher scores indicating greater likelihood of death or functional decline. Stata/SE version 12 was used for analyses (StataCorp. 2013. Stata Statistical Software: Release 13. College Station, TX: StataCorp LP).

Interviews were not recorded. Responses to the open-ended questions were transcribed verbatim and used for a qualitative analysis regarding older adults’ personal thoughts and experiences surrounding ICD decision making in the context of competing health risks. Transcripts were not returned to participants for comment and/or correction. Qualitative analysis was performed by extracting themes from the transcripts guided by our research questions. An “editing analysis” approach was used to extract themes until saturation was reached. In this method, a coding template is derived from the data itself. [23] Two investigators (AG and BL) independently reviewed all transcripts to extract preliminary categories of themes. The transcripts were reviewed iteratively to make modifications to the coding template and add new categories as needed. The study group then reviewed the findings and organized the categories into themes for presentation. Differences were discussed and resolved by consensus. Quotations that the investigators deemed to be most representative of the responses were selected for inclusion. Participants were not asked to provide feedback on the findings. Consistent with best practices on rigorously evaluating mixed methods research in the health sciences [24], we report the quantitative data in conjunction with qualitative quotes and themes that help to develop a more complete understanding of the statistical results.

## Results

### Characteristics of participants

The clinic database included 328 patients with ICDs. After excluding patients who were <70 years of age (*n* = 204), had dementia (*n* = 10), or whose devices had been inactivated (*n* = 2) or upgraded to CRT-D (*n* = 4), 108 eligible patients remained. Of those, 33 were lost to clinic follow-up, 12 did not respond to repeated phone calls, and 4 had died (Fig. 1). Fifty-nine eligible patients were contacted by RG; 44 agreed to participate (75 % response rate).

Table 1 depicts the demographic characteristics, baseline health, and VES scores of the participants. In general, participants were elderly, male, and white, with a high prevalence of comorbidities and geriatric impairments. The mean (SD) health literacy score was 2.3 (2.4) out of 12. The mean (SD) VES-13 score was 3 (2.8). Nineteen respondents (43 %) had VES-13 scores ≥3. The average (SD) length of time from initial ICD placement to the start of the study was 7.3 (3.9) years.

**Table 1:** Demographics of survey respondents

	*N* (%)
Age, mean (SD)	77.5 (5)
Sex, female	13 (29.6)
Race, white	39 (88.6)
Less than 12th grade education	16 (36.4)
Health literacy, mean (SD)^a^	2.3 (2.4)
Married	26 (59.1)
Comorbidities and geriatric syndromes, mean (SD)	6.9 (2.7)
VES-13 score, mean (SD)	3.0 (2.8)
VES-13 score ≥3	19 (43)
Years since ICD implantation, mean (SD)	7.3 (3.9)
Prior ICD shock	21 (47.7)

^a^We assigned 0 (no problems with reading) to 4 points (highest problems with reading) to the responses for each question. Scores for the 3 health literacy screening questions were combined to obtain a 12-point scale, with 0 = no problems with health literacy and 12 = highest problems with health literacy [22].

### Preferences for ICD therapy

Our primary objective was to determine whether older adults would choose to get an ICD again in their current health. Overall, 91 % of participants (40 of 44) said they would “definitely” choose to get an ICD again in their current health and 7 % (3 of 44) said they would “possibly” choose to get one again. Similarly, 98 % of participants felt that in their current health, the potential benefits of their ICD were worth the burdens.

### Qualitative findings

**Fig. 2:**
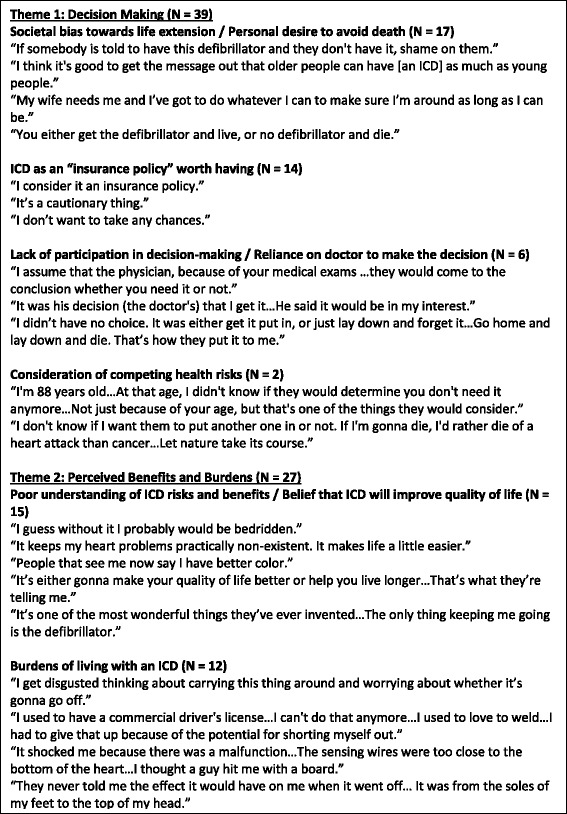
The figure depicts themes, subthemes and representative quotes that emerged as patients discussed their attitudes towards ICD implantation in the context of competing health risks

By and large, patients revealed a strong desire to extend life, expressed complete confidence in the lifesaving capabilities of their ICDs, and did not describe consideration of competing health risks. Two major themes emerged as patients discussed their attitudes towards ICD implantation in the context of competing health risks: (1) Decision making, and (2) Perceived benefits and burdens. Within the decision making theme, there were several sub-themes: (a) Societal bias toward life extension/Personal desire to avoid death; (b) ICD as an “insurance policy” worth having; (c) Lack of participation in decision making/Reliance on doctor to make the decision; and (d) Consideration of competing health risks. Within the perceived benefits and burdens theme, there were two sub-themes: (a) Poor understanding of ICD risks and benefits/Belief that ICD will improve quality of life; and (b) Burdens of living with an ICD. Representative quotes from participants are below and depicted in Fig. 2.

#### Theme 1: decision making

##### Societal bias toward life extension/Personal desire to avoid death

Patients spoke about the defibrillator in absolute terms, as if there were no uncertainty regarding the possible outcomes of ICD implantation. As one patient said, “You either get the defibrillator and live, or no defibrillator and die.” Another patient expressed a sense of moral obligation to accept the ICD and try to live as long as possible: “If somebody is told to have this defibrillator and they don’t have it, shame on them.”

##### ICD as an “insurance policy” worth having

Patients frequently referred to their ICDs as a “safeguard” or an “insurance policy.” For example, one patient stated, “I don’t want to take any chances.” Another referred to it as “a cautionary thing.”

##### Lack of participation in decision making/Reliance on doctor to make the decision

Many patients’ responses suggest that they had played a passive role in the decision of whether or not to accept an ICD. For example, one patient said, “I didn’t have no choice…It was either get it put in, or just lay down and forget it…Go home and lay down and die.” Another stated, “It was his decision (the doctor’s) that I get it…He said it would be in my interest.”

##### Consideration of competing health risks

Only two participants suggested that competing health risks might play a role in their decision of whether or not to accept an ICD. One patient, an 88-year-old woman, said, “At that age, I didn’t know if they would determine you don’t need it anymore…Not just because of your age, but that’s one of the things they would consider.” Another patient suggested that it would be preferable to die quickly of sudden cardiac death, rather than face a progressive illness: “I don’t know if I want them to put another one in or not. If I’m gonna die, I’d rather die of a heart attack than cancer…Let nature take its course.”

#### Theme 2: perceived benefits and burdens

##### Poor understanding of ICD risks and benefits/Belief that ICD will improve quality of life

A recurring theme was that ICDs would improve quality of life. For example, one patient said, “I guess without it, I probably would be bedridden,” and another stated, “It keeps my heart problems practically non-existent. It makes life a little easier.” Most patients were unaware that one can choose to accept a pacemaker for relief of symptoms, yet forego the defibrillator to avoid shocks if life extension is not a goal.

##### Burdens of living with an ICD

Twelve patients discussed the burdens of living with an ICD. For example, one man reflected, “I get disgusted thinking about carrying this thing around and worrying about whether it’s gonna go off.” Another said, “They never told me the effect it would have on me when it went off… It was from the soles of my feet to the top of my head.” Patients generally expressed the belief that the potential benefits of an ICD outweighed any burdens. However, as described above, patients infrequently weighed these tradeoffs in the decision of whether to accept an ICD.

### ICD experiences and knowledge

**Fig. 3:**
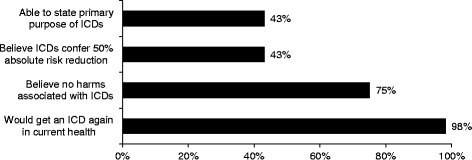
The figure depicts the percentage of patients that gave each response to questions about ICD knowledge and beliefs

Twenty-one respondents (47.7 %) had received a prior ICD shock. Of the 23 respondents who had never been shocked, 7 thought the ICD had saved their life and 10 were unsure. Thirty-three participants (75 %) thought there were no potential harms associated with their ICDs (Fig. 3). Among shock recipients, 8 (38 %) were able to name a harm associated with ICDs; these included restrictions on driving and using welding equipment, device malfunction, soreness, shocks, physical appearance, and need for device replacement in the future. Three (13 %) of the patients who had not been shocked identified a harm associated with ICDs. One participant reported an ICD-related complication (“It shocked me because there was a malfunction”). When asked about the survival benefit of ICDs, 43 % incorrectly assumed that an ICD confers a 50 % absolute survival benefit over 5 years and 41 % said “don’t know” (the survival benefit of ICDs in at-risk patients who have not survived cardiac arrest is 5 to 7 % over 5 years).^1, 2^ When asked about the primary purpose of an ICD, fewer than half (43 %) answered correctly (“To prevent sudden cardiac death”). Three participants thought an ICD would improve symptoms of heart failure and 7 said “don’t know.”

## Discussion

The major finding of this study was that older adults with multiple chronic conditions and ICDs remained enthusiastic about device implantation despite high short-term risk of death or functional decline. Nearly half overestimated the survival benefit of ICDs. The majority of patients did not consider their competing health risks in their decision of whether or not to accept an ICD, perhaps because they were unaware of their prognosis, or because of a societal bias to embrace potentially curative treatment, regardless of potential consequences.

Patients revealed considerable gaps in their understanding of ICDs. Importantly, 7 participants who had never been shocked thought the ICD had saved their life and 10 were unsure. Using a question from prior ICD survey research [21], we found that nearly half of those surveyed expected an ICD to save greater than 50 lives per 100 during 5 years. The multiple choice question presented absolute risks using frequencies, rather than relative risks. This method has been shown to improve patients’ understanding of risks and benefits, and is considered “state of the science” in shared decision making [25]. Poor grasp of complex medical information is not isolated to defibrillator technology, nor does it necessarily indicate that accurate information was not conveyed to patients at the time of implantation. A variety of factors may prevent older adults from achieving truly informed consent [26]. Marginal health literacy may be one contributing factor; the results of the health literacy screen suggest that patients in our sample performed well with simple medical tasks but may sometimes have had difficulty comprehending more complicated medical information [22]. The patients in our study were recalling discussions that happened years ago; we do not know what they were told. Previous research has suggested that cardiologists may downplay information about psychological and long-term risks when discussing ICDs with eligible patients [10, 27]. Our data do not allow us to reach conclusions about the reason for patients’ poor understanding of ICDs. In addition, we did not have information on patients’ perceived health status. It is possible that the participants considered themselves to be in relatively good health with reasonable life expectancy, though many had high VES-13 scores. This misunderstanding is a barrier to informed consent and patient-centered care.

Our findings are consistent with previous studies, which found that patients perceive an obligation to undergo cardiac interventions despite advanced age, overestimate the survival benefits conferred by ICDs, and are unwilling to deactivate their devices even when presented with hypothetical scenarios in which they are likely to die soon of cancer or another noncardiac cause [21, 28]. Our data expand upon previous qualitative studies of ICD decision-making, which were small, included patients as young as 18 years [20, 29], or examined preferences for ICD deactivation in the context of hypothetical health scenarios [20, 21]. One study found that the majority of patients (mean age 71.4 years), after hearing an informational script about the benefits and burdens of ICDs, would opt for ICD deactivation in hypothetical end-of-life scenarios such as advanced dementia or prolonged mechanical ventilation [20]. Yet it has long been recognized that “the willingness to suffer for a chance to postpone death may be felt more acutely by those nearer to death,” [30] so it is not clear how such patients would decide if in these clinical situations. Our study addressed a slightly different question, ICD placement rather than deactivation. A major strength of our study was that the patients were older and had a higher prevalence of functional impairment than patients in previous studies; 43 % of patients in our study had VES-13 scores ≥3, conferring a 50 % likelihood of further functional decline or death within 2 years.

Our findings are relevant given the large number of older adults undergoing ICD implantation [2]. The decision to implant an ICD in older adults is complex, particularly for primary prevention in patients who are at risk for sudden cardiac arrest because of systolic dysfunction but have never experienced a ventricular arrhythmia. A substantial number of older adults currently receiving ICDs may not actually benefit from them, either because they are not at high risk of sudden cardiac death or because they are more likely to die of other causes; only one-quarter of patients receive appropriate ICD shocks within 5 years [31]. Many older patients have multiple chronic conditions, increasing their risk of death from non-arrhythmic causes. Current ICD guidelines recommend against defibrillator implantation in patients with life expectancy less than 1 year [6]. Functional status measures such as those in the VES-13 are not routinely obtained during cardiology office visits [18]. Functional status falls outside the traditional disease model and is often under-recognized by non-geriatricians, though it predicts mortality and may impact the effectiveness of ICD use, as well as an older adult’s decision to accept an ICD. Older adults with multiple coexisting diseases and functional impairments may weigh the potential benefits and harms of ICD therapy differently if consent were truly informed about the benefits and harms of ICDs and the risk of competing health events [32, 33]. For those with multiple chronic conditions and poor functional status, the “rescue culture” of modern medicine can result in a spiral of aggressive therapies that may have little potential for benefit and may instead increase distress near the end of life [13, 34, 35]. The societal imperative to avoid death in frail, older patients may result in “new pathways to death and new qualms for patients and families” [13, 28].

Our findings must be interpreted in the context of several limitations, the first being survival bias. Study participants had had their ICDs for an average of 7 years. This suggests that these patients were appropriately selected for ICD therapy, because they did not succumb to competing health risks in the first few years after implantation. Their responses may have been biased because they had done well with their ICDs. However, we asked patients if they would choose to get an ICD again in their *current* health (irrespective of their baseline health status). These patients were willing to undergo ICD placement again, despite the fact that many had poor functional status at the time of the study. This is important because many older adults with ICDs will survive long enough to consider generator replacement, and their health status may have declined since the initial implant. A decision that may have been relatively straightforward in the past may become more complex as an older adult’s health status changes, and clinicians need to be attuned to this when counseling patients and families about ICDs.

The second limitation is that only 44 of 104 eligible subjects (excluding 4 who had died) completed the survey. The 33 patients who were lost to clinic follow-up and 12 who did not respond to repeated phone calls were likely different than the patients who agreed to participate. Patients who declined ICDs, no longer followed up with the electrophysiology clinic, or died from ICD complications or comorbidities likely have negative viewpoints that we did not document. Only one participant had experienced an ICD-related complication. Therefore, the generalizability of our study to the broader population of older adults with ICDs is uncertain. Despite these caveats, our findings are important because they reveal a high degree of misunderstanding about the potential benefits and harms of ICDs.

The third limitation is that we were unable to distinguish between patients who received their ICDs for primary or secondary indications. Patients’ attitudes may differ depending on the indication for ICD therapy. Fourth, our patients were recruited from a single academic cardiac device clinic. Their experiences may not be representative of patients at other institutions. Although we did not formally assess the rate of periprocedural or late (≥90 days) complications among the study population, we estimate that the rate at our institution is consistent with that reported in the literature [7]. Patients at referral centers may be more likely to accept treatment with invasive medical technologies [21]. Fifth, the investigator who made the initial contact with potential participants is well-known to them because he conducts all of their device interrogations, and this may have positively influenced their perspectives toward ICD therapy. Patients’ acceptance of ICDs is strongly influenced by cardiologists’ opinions [29]. Sixth, our data did not permit us to determine the timing of ICD shocks. Patients may feel heightened anxiety and regret about their ICD in the immediate post-shock period. Seventh, the small sample size limits generalization.

A final limitation is that some of the patients had pacemaker-defibrillators and may have conflated the two separate functions of the device. For example, the patient who said, “The only thing keeping me going is the defibrillator” may have had severe sinus node dysfunction that improved after receiving a pacemaker-defibrillator. However, our consent script and questions explicitly stated that the study was about defibrillators. Many patients have indications for pacemakers and defibrillators, and they should understand the distinction so that they can make an informed choice. An ICD itself does not improve quality of life. A patient with poor functional status and limited life expectancy due to other comorbidities may want a pacemaker but wish to forego the possibility of defibrillator shocks.

Despite these limitations, this study expands previous literature and raises important concerns about the quality of ICD decision making for older adults with competing health risks, and substantial patient misunderstanding regarding the potential benefits and harms of ICD therapy. Although the nation’s leading cardiology and heart failure organizations have called for shared decision making regarding cardiac implantable electronic devices and consideration of prognosis, physical function and quality of life, cardiologists receive little if any formal training in how to conduct such conversations with patients about whether or not to pursue treatment with invasive technologies [36–38]. Furthermore, physician quality metrics and professional society guidelines may serve as barriers to patient-centered care by heavily influencing cardiologists’ decision making regarding ICDs [29, 39]. As a result, clinicians may be reinforcing patient misperceptions, and the care patients receive may frequently not be the care they or their families would choose if properly informed [21, 32, 40]. Decision aids are a potential solution to improve ICD decision making [41]. A recent systematic review identified 4 decision aids for patients considering ICD insertion and found that the tools “included comprehensive content on technical aspects of insertion, but made limited references to implications for quality of life and generally lacked balance in terms of how the decision to insert was presented.” [42] The development of high-quality decision aids that address not only the technical aspects of ICD insertion, but also issues related to how ICDs may affect quality of life, is needed.

Incorporation of comprehensive geriatric assessment has been recommended within the fields of geriatric oncology and nephrology in order to identify risk factors for increased vulnerability and inform treatment decisions of older patients with cancer and end-stage renal disease [43, 44]. Similarly, awareness of geriatric impairment among older adults who are eligible for ICDs could lead to improved clinical decision making.

## Conclusions

Our primary finding was that most older adults with ICDs do not weigh their competing health risks and life expectancy in ICD decision making. This suggests the need to develop innovative strategies for incorporating non-disease specific assessment of prognosis in discussions with ICD-eligible patients, and to partner more effectively with patients and families to decide how best to use medical technologies, particularly for older adults with competing health risks.
